# Body mass index is associated with reduced exhaled nitric oxide and higher exhaled 8-isoprostanes in asthmatics

**DOI:** 10.1186/1465-9921-8-32

**Published:** 2007-04-16

**Authors:** Sushma Komakula, Sumita Khatri, Joel Mermis, Samira Savill, Shireen Haque, Mauricio Rojas, LouAnn Brown, Gerald W Teague, Fernando Holguin

**Affiliations:** 1 Department of Medicine, Emory University, Atlanta, USA; 2 Department of Medicine, Case Western, Ohio, USA; 3 Department of Pediatrics, Emory University, Atlanta, USA; 4 Davis-Fischer Building, 550 Peachtree Street, NE, 2^nd ^Floor, Room 2331, Atlanta GA 30308, USA

## Abstract

**Background:**

Recently, it has been shown that increasing body mass index (BMI) in asthma is associated with reduced exhaled NO. Our objective in this study was to determine if the BMI-related changes in exhaled NO differ across asthmatics and controls, and to determine if these changes are related to increased airway oxidative stress and systemic levels of leptin and adiponectin.

**Methods:**

Observational study of the association of BMI, leptin, and adiponectin with exhaled nitric oxide (NO) and exhaled 8-isoprostanes in 67 non-smoking patients with moderate to severe persistent asthma during baseline conditions and 47 controls. Measurements included plasma levels of leptin, adiponectin, exhaled breath condensates for 8-isoprostanes, exhaled NO, pulmonary function tests, and questionnaires regarding asthma severity and control.

**Results:**

In asthmatics, BMI and the ratio of leptin to adiponectin were respectively associated with reduced levels of exhaled NO (β = -0.04 [95% C.I. -0.07, -0.1], p < 0.003) and (β = -0.0018 [95% C.I. -0.003, -0.00034], p = 0.01) after adjusting for confounders. Also, BMI was associated with increased levels of exhaled 8-isoprostanes (β = 0.30 [95% C.I. 0.003, 0.6], p = 0.03) after adjusting for confounders. In contrast, we did not observe these associations in the control group of healthy non-asthmatics with a similar weight distribution.

**Conclusion:**

In adults with stable moderate to severe persistent asthma, but not in controls, BMI and the plasma ratio of leptin/adiponectin is associated with reduced exhaled NO. Also, BMI is associated with increased exhaled 8-isoprostanes. These results suggest that BMI in asthmatics may increase airway oxidative stress and could explain the BMI-related reductions in exhaled NO.

## Background

In recent years, there has been a parallel increase in the prevalence of both asthma and obesity. This has led to the speculation that increased BMI is a risk factor for asthma [1]. Several cross sectional studies have shown higher odds for developing asthma among obese children and adults [2-5]; however, these studies were limited by their inability to address the direction of this association, and were susceptible to confounding factors [6]. Prospective studies have shown that increasing BMI antedates the diagnosis of asthma incidence and bronchial hyperresponsiveness. This temporal pattern supports the idea that obesity may actually cause versus simply associate with asthma [7-11]. Further, obese asthmatics are also at increased risk of having more severe respiratory symptoms, increased emergency room visits, and poor asthma control [5,12-16]. The mechanisms by which obesity increases asthma incidence or increases disease severity in asthmatics are unknown.

Recent experimental data suggest that obesity-related changes in adipokines could play a critical role in mediating airway inflammation and bronchial hyperresponsiveness (BHR) [6]. Leptin, an adipokine elevated in obesity and known to induce satiety, has been shown to up-regulate various cytokines, promoting a state of chronic inflammation [17]. Compared to saline treated mice, leptin-infused mice had higher systemic IgE levels and increased bronchial hyperresponsiveness only after ovalbumin (OVA) inhalation [18]. In contrast, adiponectin, which is reduced in obesity, and has anti-inflammatory and antioxidant properties [19-21], has been shown to reduce BHR and decrease airway inflammation following OVA inhalation in mice [22]. However, whether or not leptin and/or adiponectin can affect exhaled NO (nitric oxide) levels and measures of airway oxidative stress in asthmatics are unknown.

In a recent study, exhaled NO was inversely correlated with BMI in subjects with asthma, yet a potential explanation for this finding was not offered [23]. The purpose of this study was to examine the association between BMI and adipokines (leptin and adiponectin) with exhaled NO in asthmatics and healthy controls, and to determine whether BMI-related changes in exhaled NO can be attributed to changes in airway oxidative stress by measuring exhaled 8-isoprostanes.

## Methods

This study was conducted at Grady Memorial Hospital in Atlanta, Georgia with the approval of the institutional review board. Inclusion criteria included: participants 18 to 70 years of age, who were previously diagnosed with moderate to severe persistent asthma (Global Initiative for Asthma (GINA) class III – IV) [24] requiring treatment with inhaled corticosteroids, ≥ 12% post-bronchodilator increase in FEV_1 _(Forced exhalation volume in one second) and a post-bronchodilator FEV_1_/FVC (Forced vital capacity) ratio greater than 0.70. Exclusion criteria included: current smokers, ex-smokers who stopped smoking at least one year prior to study enrolment, or total-life smoking history > 10 pack-year, and evidence of other lung diseases or any other significant non-pulmonary co-morbidities such as congestive heart failure with ejection fraction < 50%, stable angina, chronic renal failure with serum creatinine > 2.0, documented cirrhosis, other disorders requiring steroid treatment (vasculitis, lupus, or rheumatoid arthritis), advanced cancer, or AIDS. Subjects were also excluded if they had an asthma exacerbation during the month preceding enrolment.

## Controls

From the hospital personnel, we recruited healthy, non-asthmatics without any history of allergic diseases. The controls were not active smokers, and met the same smoking exclusion criteria used for the study population. Controls were selected to match the gender, race, and weight distribution of the study population.

## Measurements

Height, weight, and waist to hip ratios were obtained in all the participants. Participant's current level of asthma severity was classified according to the 2004 GINA guidelines into mild intermittent (Class I), mild persistent (Class II), moderate persistent (Class III), and severe persistent (Class IV) asthma. Participants also completed the Juniper asthma control questionnaire (ACQ)[25]. Atopy was based on a positive or negative skin prick test documented in the clinic records. In all patients, obstructive sleep apnea (OSA) and gastro-esophageal reflux disease (GERD) were ascertained by either medical history or use of medications to relieve GERD symptoms.

Exhaled NO was determined using an on-line continuous chemiluminescence analyzer (CDL 88 sq Michigan, USA) after overnight fasting following the American Thoracic Society guidelines prior to spirometry [26]. Exhaled breath condensate (EBC) was collected using the Rtube, a non-invasive breath condensate collection device (Charlottesville, VA). To produce the condensate we used a metal sleeve at an initial temperature of -10 Celsius, and instructed subjects to breathe normally for 15 minutes while using a nose-clip. After the collection session, the samples were immediately stored in -70°C until analyzed. Exhaled 8-isoprostanes were determined in duplicate using an immunoassay (Cayman Chemical, Ann Arbor, MI, USA) [27]. Pre and post-bronchodilator spirometry were determined according to ATS guidelines [28]. Functional residual capacity (FRC) was done using a nitrogen gas dilution technique (CDL 88 sq Michigan, USA).

In overnight fasting blood samples, we determined levels of leptin and adiponectin. Adipokine levels were determined in duplicate using Luminex analysis (Linco research Inc., St. Charles Missouri) [29].

## Analysis

We used the Kruskal Wallis to compare non-normally distributed variables across BMI categories, and a t-test to compare means between asthmatics and controls. The chi square statistic was used to test the distribution of categorical variables. We modelled the association of BMI and adipokines (leptin, adiponectin, the leptin/adiponectin ratio) with the log-normal of exhaled NO or exhaled 8-isoprostanes using multivariable regression analysis. For the asthma subjects, we adjusted the model for the following potential confounders: age, gender, atopy, mean Juniper scores, degree of airflow obstruction (FEV_1_/FVC), diagnosis of GERD, use of long-acting beta agonists, and anti-leukotriene drugs. Given that all subjects with asthma were taking inhaled corticosteroids, this medication was not adjusted for in the model. Regression models were checked for influential points, collinearity and distribution of residuals. Statistical analysis was done using SAS 9.1 (Cary, NC), a p< 0.05 was considered significant.

## Results

Sixty-seven patients were recruited from the Asthma and Allergy Clinic at Grady Memorial Hospital in Atlanta, Georgia. The characteristics of the study population are shown in table 1.

**Table 1:** Characteristics of the study population

N	67
Average age in years (Range)	48.9 (18 – 69)
Gender (% female)	83
White (%)	6
Black (%)	81
Other (%)	13
Ex-smokers n (%)	21 (33%)
Average Pack-per-year (95% C.I.)	4 (2 – 5)
Average body weight in pounds (95% C.I.)	198 (186 – 209)
Average BMI (95% C.I.)	32 (31 – 35)
Normal weight (BMI ≥ 18 ≤ 25) (%)	14
Overweight (BMI > 25 < 30)	28
Obese (BMI ≥ 30)	58
Average waist/hip ratio (95% C.I.)	0.87 (0.85 – 0.9)
Diabetes (%)	18
GERD treatment (%)	60
Hypertension (%)	45
Obstructive sleep apnea (%)	12
Average FEV_1 _(95% C.I.)	2.1 (2.0 – 2.3)
Average FVC (95% C.I.)	2.8 (2.6 – 3.0)
Average FEV_1_/FVC (95% C.I.)	0.80 (0.7 – 0.8)
Average FRC (95% C.I.)	3.2 (2.8 – 3.5)
Average Exhaled NO (95% C.I.) (ppb)*	25 (19 – 30)
Average Exhaled 8-isoprostanes (95% C.I.) (pg/ml)	11 (9.6 – 12.4)
Average Adiponectin (μg/ml) (95% C.I.)	3 (2.9 – 3.4)
Average Leptin (ng/ml) (95% C.I.)	15 (12 – 18)

BMI (Body mass index), FEV_1 _(Forced exhalation volume in one second), GERD (Gastro esophageal reflux disease), FRC (Functional Residual Capacity), Plasma adipokines were available for 50 asthmatics. Exhaled 8-isoprostanes were available for 56 asthmatics.

The majority of participants were females, African American, obese, and 70% had a positive skin test for allergens documented in the clinic charts. Approximately a third of the population were ex-smokers, although the average amount of pack-years smoked was only 4. None of the patients had evidence of significant chronic airway obstruction. The mean post-bronchodilator FEV_1 _and FVC percent predicted were respectively 73% (95% C.I. 70 – 79) and 81% (95% C.I. 75 – 86).

### Clinical measures of asthma severity and control

**Figure 1:**
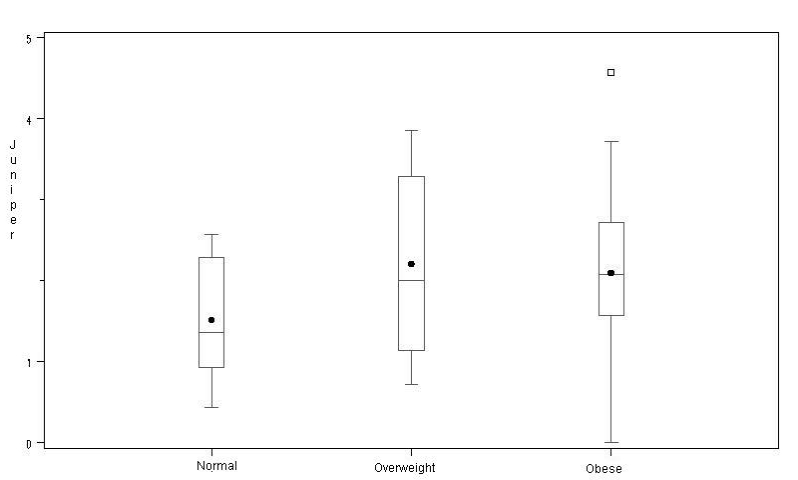
Distribution of asthma Juniper control scores by body mass index category.

**Figure 2:**
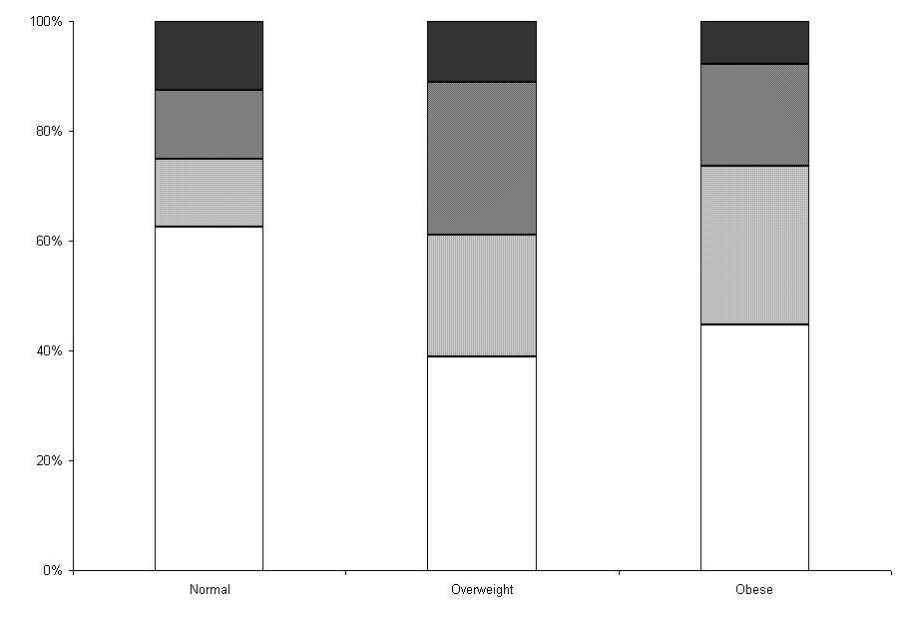
**Global Initiative for Asthma scores for asthma severity by body mass index**. [See additional file: figure keys].

The average Juniper score for asthma control was 2 (95% C.I. 1.8 – 2.3). Although we observed a trend for higher Juniper scores in overweight and obese subjects with asthma, this difference was not statistically significant (p = 0.2) (Figure 1). Moderate or severe persistent asthma, based on the GINA score, was not more prevalent in obese vs. non-obese participants (overall association of GINA scores across BMI categories, *p *= 0.8) (Figure 2). All asthmatics were taking an inhaled corticosteroid on a daily basis; other medications included: 61% long-acting β-agonists, 10% inhaled anti-cholinergics, 53% leukotriene receptor blockers, 91% regular use of short acting β-agonists, and 30% were taking combined therapy with long-acting β-agonists and leukotriene receptor blockers.

### Association of BMI with exhaled NO and exhaled 8-isoprostanes

**Figure 3:**
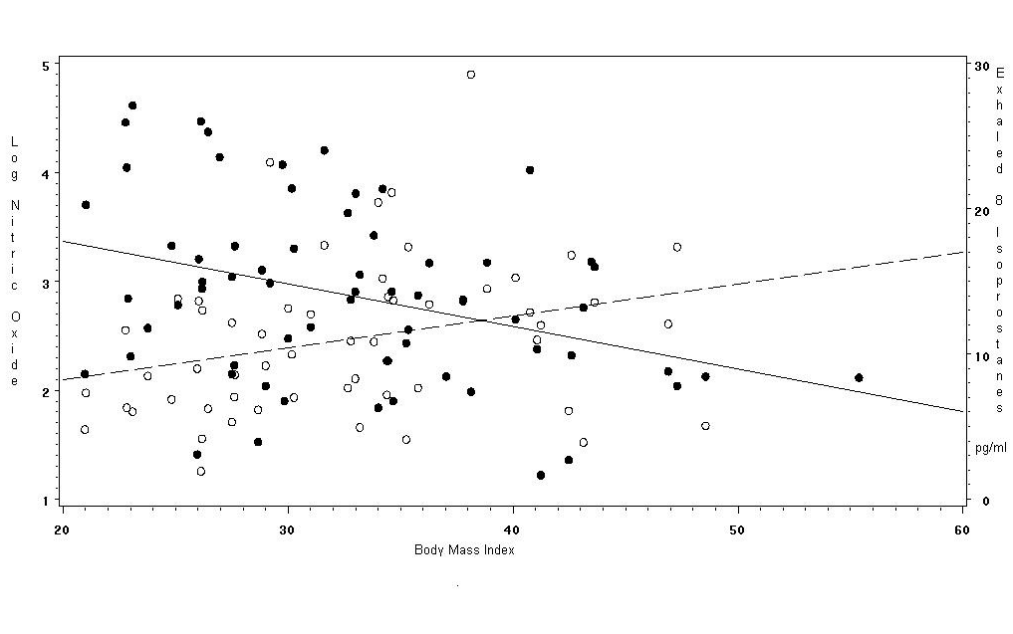
**Association of exhaled nitric oxide and exhaled 8-isoprostanes by body mass index in adults with asthma**. [See additional file: figure keys].

BMI was inversely associated with exhaled log-NO in univariate (β = -0.04 [95% C.I. -0.06, -0.1; p < 0.003]) and multivariate analysis (β = -0.04 [95% C.I. -0.07, -0.1; p < 0.003]), after adjusting for confounders. The correlation between BMI and log-NO was *r*^2 ^= -0.35 (p < 0.01). Also, BMI was associated with increased exhaled 8-isoprostanes, in univariate (β = 0.22 [95% C.I. 0.04, 0.7; p = 0.03]) and multivariate analysis (β = 0.30 [95% C.I. 0.003, 0.6; p = 0.03]) after adjusting for the same covariates. The correlation between exhaled 8-isoprostanes and BMI was *r*^2 ^= 0.33 (p = 0.03) and for exhaled 8-isoprostanes and log-NO the correlation was *r*^2 ^= -0.16 (p = 0.2). Figure 3 illustrates the association of log-transformed NO and exhaled 8-isoprostanes with BMI in the asthmatics.

### Association of leptin and adiponectin with exhaled NO and exhaled 8-isoprostanes

There were no significant associations between leptin or adiponectin with exhaled log-NO; however, the ratio of leptin (ng/ml) to adiponectin (μg/ml) (mean 0.0049 [95% C.I. 0.003–0.005]) was significantly associated with exhaled log-NO in univariate (β = -0.00035 [95% C.I. -0.002, -0.0003; p = 0.04]) and multivariate (β = -0.0018 [95% C.I. -0.003, -0.00034; p = 0.01]) analysis adjusting for confounders. The correlation between the leptin/adiponectin ratio and the log-NO was *r*^2 ^= -0.28 (p = 0.04). We did not observe significant associations between leptin, adiponectin, and their ratio with exhaled 8-isoprostanes.

### Comparison between asthmatics and the control population

A total of 47 controls were recruited for the study. Their average age was 40 years (Range: 20 – 62) and was lower than in the asthmatics (p < 0.01). The mean weight was 186 lb (95% C.I. 174 – 198) and 51% were obese, 78% were female, and 88% were African American. These values did not statistically differ from the study population. Compared to the asthmatics, controls had lower exhaled NO levels (13 ppb [95% C.I. 10–15])(p< 0.01), higher FEV_1 _(2.7 L [95% C.I. 2.5–3]), FVC (3 L [95% C.I. 2.8–3]) and FEV_1_/FVC ratio (0.86 [95% C.I. 0.8–0.9]), and similar FRC (2.9 L [95% C.I. 2.5–3.3]). The levels of leptin (16 ng/ml [95% C.I. 12–20]) and adiponectin (31 μg/ml [95% C.I. 28–35]) did not differ across groups, nor did the levels of exhaled 8-isoprostanes (11 pg/ml [95% C.I. 8–13.8]).

**Figure 4:**
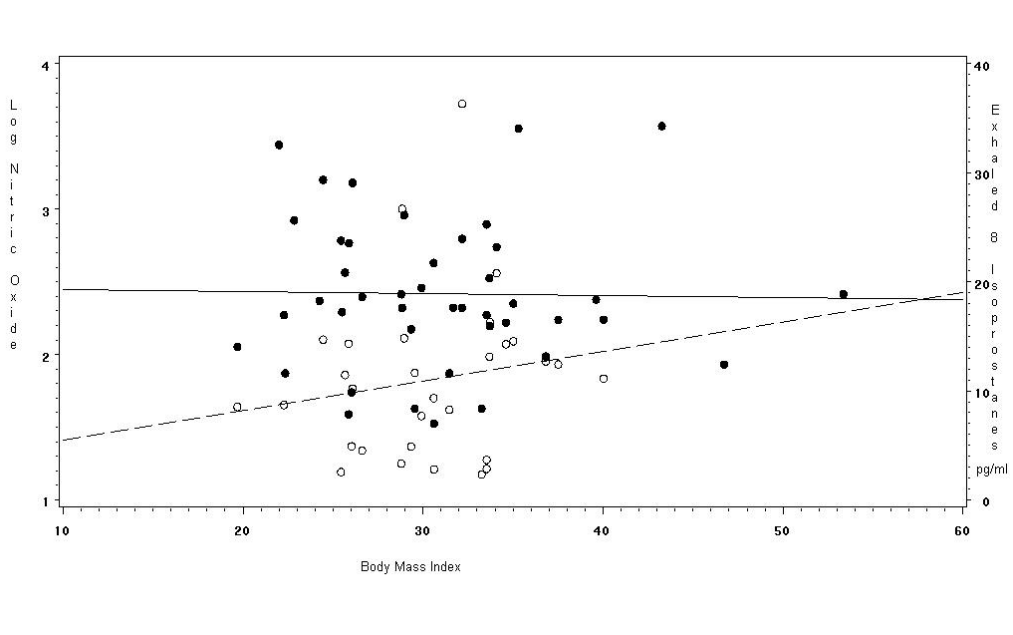
**Association of exhaled nitric oxide and exhaled 8-isoprostanes by body mass index in healthy non-asthmatic adults**. [See additional file: figure keys].

**Figure 5:**
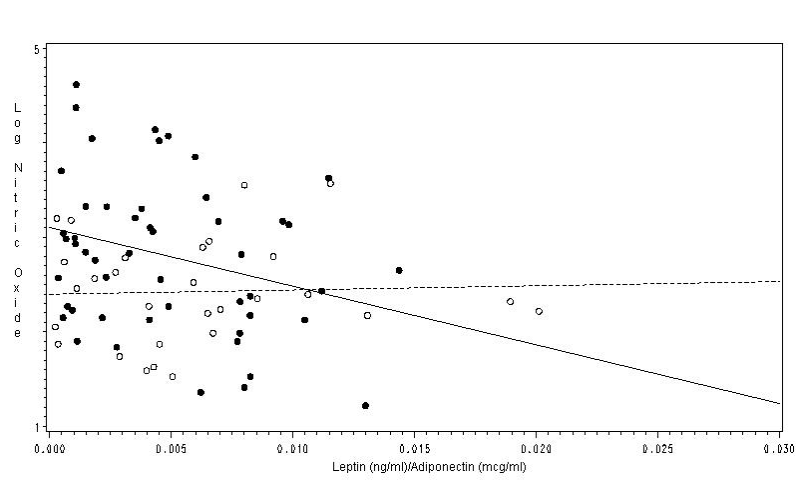
**Association of the ratio of serum leptin and serum adiponectin with the log of exhaled NO in subjects with asthma and controls**. [See additional file: figure keys].

In the controls, there was no significant association between BMI with the log of exhaled NO (β = -0.001 [95% C.I. -0.02, 0.02; p = 0.9]), and exhaled 8-isoprostanes (β = 0.27 [95% C.I. -0.33, 0.88; p = 0.3]); also, there was no significant association between the ratio of leptin/adiponectin ratio with exhaled NO (β = -0.001 [95% C.I. -0.02, 0.02; p = 0.9]). Figure 4 illustrates the linear association of log-transformed NO and exhaled 8-isoprostanes with BMI in the controls. The linear association of the leptin/adiponectin ratio in subjects with asthma and the controls is shown in Figure 5.

## Discussion

This study evaluated the association of BMI and systemic levels of leptin and adiponectin with levels of exhaled NO and exhaled 8-isoprostanes in subjects with stable asthma and healthy controls. In subjects with asthma, BMI and the systemic leptin/adiponectin ratio were independently associated with a reduction in exhaled NO, and BMI was associated with increased levels of exhaled 8-isoprostanes. In contrast, these associations were not observed in healthy controls with a similar weight distribution. To our knowledge, this is the first study describing the association between BMI, leptin and adiponectin with exhaled NO and exhaled 8-isoprostanes in adults with asthma and in healthy controls.

Although there is compelling epidemiological evidence to support an association between obesity and asthma, plausible mechanisms for this association remain poorly understood. It has been proposed that either obesity-related changes in adipokines and/or the chronic systemic inflammation in obesity, could lead to a parallel increase in airway inflammation. Exhaled NO, a sensitive biomarker of airway inflammation in asthma, would therefore be expected to be higher in obese versus non-obese asthmatics [6]. However, studies on BMI and exhaled NO do not clearly support this assertion. Some studies have found a positive correlation between BMI and exhaled NO in healthy adults [30,31], whereas others have reported no differences between obese and non-obese asthmatic children [32]. In contrast, our results showed that exhaled NO was inversely associated with BMI, after adjusting for potential confounders in stable asthmatics. Our results are similar to the findings from Barros et al, that showed a negative association between BMI and exhaled NO (*r*^2 ^= -0.32 vs. *r*^2 ^= -0.35 in our study) in 297 non-smoking asthmatics with a mean BMI of 26 (95% C.I. 25.4 – 26.5) after controlling for potential confounders [23]. The negative association between BMI and exhaled NO does not necessarily imply that increasing BMI leads to less airway inflammation; it could imply however, that increasing BMI could lead to changes in baseline airway NO redox metabolism, through an increase in baseline airway oxidative stress. In the presence of increased reactive oxygen species, airway NO can be readily converted into reactive nitrogen species (RNS) [33]. Because the total measured exhaled NO is the end product of NO produced – NO consumed, an increase in the RNS/NO ratio would result in lower measured exhaled NO levels [34].

Our data support this hypothesis by showing a significant association of BMI with exhaled 8-isoprostanes. Studies have shown that asthmatics have higher levels of exhaled 8-isoprostanes than non-asthmatics, and that levels increase with asthma severity [35-37]. In addition, BMI has been associated with higher levels of plasma and urinary levels of 8-isprostanes in men and women [38,39]. In contrast to the inverse association between exhaled 8-isoprostanes and exhaled NO observed in our study, Montushi et al reported a positive correlation [37]; however, this correlation was observed in only 12 mild asthmatics that were not on inhaled corticosteroids, and was not reported for patients with more severe disease that were on inhaled corticosteroids; further, no data on body weight was provided. It is possible that the association between exhaled 8-isoprostanes with exhaled NO changes, depending on whether or not this association is determined during an asthma exacerbation or during baseline conditions.

Alternatively, increasing BMI may lead to an increase in airway oxidative stress via obesity-related changes in adipokines. For example, Leptin increases proportionately with BMI and has been shown to produce reactive oxygen species (ROS) [40,41] through multiple mechanisms including, endothelin-1 receptor activation, reduced Nicotine Adenine Dinucleotide (NAD(p)H) oxidase activation, and production of Tumor Necrosis Factor alpha (TNF-α) [42-44]. Further, leptin levels in the bronchoalveolar lavage fluid are increased in obese mice models, and instillation of leptin in the airway is associated with acute lung injury in the presence of hyperoxia [45]. In contrast, adiponectin is inversely associated with biomarkers of inflammation and with BMI [19,46], and low levels of adiponectin have been associated with increased systemic oxidative stress, and reduced NO production from endothelial cells [47,48].

It is possible that the amount of oxidative stress necessary to shift the airway redox balance towards conversion of airway nitric oxide into RNS exists only when there is a certain balance of leptin and adiponectin. For example, obesity leads to increased leptin and reduced adiponectin; this obesity-induced state of hyperleptinemia and hypoadiponectinemia is associated with increased systemic inflammation and oxidative stress [46,49]. It is therefore possible that reaching a certain threshold of obesity-related systemic oxidative stress also results in increased airway oxidative stress. In our study, the ratio of leptin to adiponectin was not associated with exhaled levels of 8-isoprostanes, which may be indicative that other adipokine-independent pathways exist in increasing BMI-related airway oxidation.

This study has some important limiting features. First, the majority of our study population was African American, female, either overweight or obese, and previously diagnosed with moderate to severe disease. These characteristics may limit the external validity of our study, particularly since women appear to be more susceptible to obesity-mediated increase in asthma severity or asthma incidence [1]; further, these results may not apply to asthmatics with milder forms of asthma severity. Second, determination of causation is impossible and determination of specific mechanisms is difficult in an observational study design. Our results do provide a mechanistic hypothesis by which obesity relates to asthma; however, these results cannot provide information as to why obesity increases the risk for asthma incidence. Third, using questionnaires to evaluate conditions such as obstructive sleep apnea and GERD has a lower sensitivity; therefore, our results could be affected by residual confounding from these misclassified co-morbid conditions. We have to also consider that un-measured confounders, including a more detailed assessment of glycemic control might have important effects on the magnitude of both systemic and airway oxidative stress. Fourth, due to the fact that asthmatics were on several inhaled medications, it is difficult to determine the extent these medicines affected our results. Though all asthmatics were on inhaled corticosteroids, the various dosages and the variable effects they may have on individual patients may confound our results. However, in the study by Barros et al [23], the association between BMI and exhaled NO was not attenuated when adjusting for inhaled corticosteroids. Further, in the 4-state U.S. National Asthma Survey (NAS) the proportion of asthmatics using ICS is not higher among the obese. In the NAS the use of inhaled corticosteroids among 1059 normal weight asthmatics was 28%, compared to 30% in 985 overweight and 34% in 1015 obese subjects with asthma (p = 0.09) (unpublished observation) [50]. Fifth, plasma adipokines and exhaled 8-isoprostane levels were only available in 50 and 56 out of 65 patients respectively, and could be a potential source of bias; however, we would expect this bias to be small if any, as the absence of these biological samples was not systematic, and was a consequence of random patient refusal. Sixth, although obese and overweight subjects had higher mean Juniper ACQ, we were not able to detect meaningful differences in asthma severity across BMI categories, given the size of patient population; however, our intention was to minimize differences in asthma severity across BMI categories, to assure that the association between BMI and airway biomarkers were not biased by differences in asthma severity. Seventh, our study is limited by determining airway oxidation stress using 8-isoprostanes using a commercial EIA kit which, although this method is highly specific and has been validated by gas chromatography, there have been contradictory results in the reproducibility of this essay [51].  Further; we did not explore other airway biomarkers of inflammation and reactive nitrogen species. Finally, we did not assess directly the atopy status in the controls, and are therefore unable to determine how underlying atopy affected the comparison of NO across adults with and without asthma.

## Conclusion

In summary, this study shows that in asthmatics, in the absence of an exacerbation, BMI and the leptin/adiponectin ratio are associated with reduced exhaled NO, and BMI is associated with increased exhaled 8-isoprostanes, possibly reflecting an increase in baseline airway oxidative stress. It remains to be elucidated whether these BMI-related changes in airway oxidation, which were determined during baseline conditions, can be associated with increased bronchial hyperresponsiveness and increased asthma severity. Because these associations were not observed among the controls, our results suggests that BMI alone is not sufficient to produce airway changes in airway NO and airway oxidative stress. Overall, these findings provide important hypothesis-generating information in understanding how obesity associates with asthma severity.

## Competing interests

Dr. Holguin has a grant from Critical Therapeutics.
